# Biomechanical Properties of Silk-Derived 3D Bioprinted Scaffolds for Cartilage Regeneration: A Comprehensive Review

**DOI:** 10.3390/biomimetics11080575

**Published:** 2026-08-12

**Authors:** Sanjana Challa, Alynah J. Adams, Laken Anderson, Bria Fiebiger, Rikin Soni, Athena Ye, Jocelyn Hunt, Charlotte Thomas, Dorien I. Schonebaum, Jose A. Foppiani, Umar Choudry, Samuel J. Lin

**Affiliations:** 1 Division of Plastic Surgery, Beth Israel Deaconess Medical Center, Harvard Medical School, Boston, MA 02215, USA; 2 School of Medicine, Medical College of Wisconsin, Milwaukee, WI 53226, USA; 3 School of Medicine, University of North Carolina at Chapel Hill, Chapel Hill, NC 27599, USA; 4 Division of Plastic and Reconstructive Surgery, University of Minnesota, Minneapolis, MN 55455, USA

**Keywords:** cartilage regeneration, cocoon silk, silk fibroin, silk bioink, 3D bioprinting, cartilage repair, mechanical properties, scaffolds, hydrogels, tissue engineering

## Abstract

Cartilage regeneration remains a major clinical challenge because cartilage has limited healing capacity and must withstand substantial mechanical loading. Silk fibroin, a cocoon-derived biomaterial with tunable mechanical properties, has emerged as a promising scaffold material for cartilage tissue engineering. This systematic review evaluates the biomechanical performance, fabrication approaches, and regenerative potential of silk fibroin bioprinted scaffolds for cartilage repair. A systematic review was conducted according to PRISMA guidelines. Primary outcomes included scaffold mechanical properties relevant to cartilage function. Secondary outcomes included scaffold composition, fabrication methods, biological performance, clinical applications, and reported limitations. Of 918 identified records, 24 studies published between 2014 and 2025 met the inclusion criteria. Most were preclinical studies using silk fibroin-based hydrogels, often combined with hyaluronic acid, gelatin, collagen, or synthetic polymers. Mechanical properties varied according to scaffold composition and crosslinking methods. Reinforced scaffolds generally demonstrated improved stiffness, structural support, and load distribution. Silk fibroin scaffolds consistently supported chondrogenesis and cartilage-like tissue formation. However, challenges remain, including mechanical mismatch with native cartilage, construct instability, cell loss, and suboptimal degradation profiles. Overall, silk fibroin bioprinted scaffolds demonstrated support for chondrogenesis and cartilage-like tissue formation in preclinical studies for cartilage regeneration. Future research should focus on standardized biomechanical testing, long-term in vivo evaluation, and clinically relevant scaffold designs to facilitate translation toward functional cartilage repair.

## 1. Introduction

Silk fibroin (SF), the principal structural protein of *Bombyx mori* silk, has a long history of clinical use and represents one of the earliest materials used in biomedical projects. The most popular use was for surgical sutures due to its strength and biomechanical characteristics [1,2]. In modern research, silk fibroin has gained attention due to its favorable properties, such as its biocompatibility, controllable biodegradation, and versatility.

Silk fibroin has several advantages that make it a biomaterial of interest for translational medicine. First, it is cost-effective and sustainably derived due to its natural abundance [1,3]. It can also be produced in large quantities, which has made it used across multiple biomedical applications. The advances in silk fibroin processing have already supported the development of resorbable devices, tissue scaffolds, and drug delivery systems across many clinical disciplines [4,5]. Despite these advances, challenges to large-scale production remain. Characterizing long-term biodegradation and implementation into human patients highlights opportunities for continued research.

Articular cartilage injury remains a major challenge due to the mechanical demands of the joint environment. Current treatment strategies have fallen short due to incomplete defect integration or limited long-term durability, especially in younger and active patients [6]. As a result, there is growing interest in the field of research around biomaterial-based approaches that can support chondrogenesis while also withstanding joint loading. Studies have shown that scaffold-based approaches can address these limitations by combining biological signals with structures that also provide mechanical support [7].

While several reviews have examined silk fibroin as a biomaterial for tissue engineering, prior works have largely focused on general bioink formulation strategies or broad overviews of scaffold design without systematically evaluating the biomechanical performance of 3D bioprinted silk fibroin constructs specifically for cartilage regeneration. To our knowledge, this is the first systematic review to comprehensively assess and compare the mechanical properties, fabrication approaches, and regenerative outcomes of silk fibroin-based bioprinted scaffolds within the context of cartilage repair. By synthesizing key outcomes, including compressive behavior, viscoelastic properties, degradation profiles, and biological performance, this review aims to identify trends across processing methods and material compositions, improve consistency within the literature, and provide a framework to guide the translation of silk fibroin-based constructs toward clinically viable cartilage repair.

## 2. Materials and Methods

### 2.1. Systematic Review

#### 2.1.1. Information Sources

A detailed systematic literature review was conducted in July 2025, following the Preferred Reporting Items for Systematic Reviews and Meta-Analyses extension for Systematic Reviews (PRISMA-ScR) guidelines. The databases searched included MEDLINE via PubMed, Web of Science, and Cochrane Central Register. PRISMA flow diagram illustrating the identification, screening, eligibility assessment, and inclusion of studies in this review is available in the Supplementary Materials (Figure S1). Ethical review and approval were waived for this study because the included studies did not involve human subjects.

#### 2.1.2. Search Strategy

The search strategy was designed by an experienced librarian using terms, subject headings, and keywords related to silk, biomaterial, bioengineering, biology, clinical outcomes, mechanical properties, physical properties, biomedical application, fibroin, wound dressing, tissue scaffolds, drug delivery, biocompatibility, biodegradability, medical therapy, and medical application.

#### 2.1.3. Eligibility Criteria

Eligibility criteria included studies of 3D-printed silk derivatives for medical applications that addressed our primary and secondary outcomes, observational studies, and clinical trials published in English, French, or Spanish. Editorials, reviews, meta-analyses, commentaries, conference abstracts, and letters were excluded because they did not report original research. Studies that did not evaluate silk-based materials, lacked mechanical property data, were ongoing, or used cadaveric models were also excluded. In addition, studies reporting treatments that did not use silk, articles lacking mechanical property data, and ongoing and cadaveric studies were excluded. Ongoing studies are unable to provide finalized results, and cadaveric studies have inherent limitations in translating findings to living subjects.

#### 2.1.4. Selection Process

The online systematic review program, Covidence (Covidence systematic review software, Veritas Health Innovation, Melbourne, Australia), was used to upload the search results [8]. Two independent reviewers conducted a two-stage screening process for study selection. First, records were screened by title and abstract. The same reviewers then independently assessed the full texts of potentially eligible articles. All discrepancies were resolved by a third researcher, who moderated discussions until a joint decision was reached. The protocol for this systematic review was registered on PROSPERO under the identification number CRD420261304371.

#### 2.1.5. Data Extraction and Outcomes

Using predesignated variables, five reviewers (A.Y, B.F, L.A, R.S, J.H) extracted the data from the final 24 articles. These variables included the authors’ names, publication month and year, journal title, country or region of study, type of study (in vivo, in vitro, or in situ), model organism or synthetic material utilized, production method, component of the cocoon silk fiber, primary and variable secondary outcomes, test models (rabbit, mouse, human, or pig), and various physicochemical and structural silk characterization measures (scaffold size, molecular weight, hydrophilicity, etc.).

We primarily sought outcomes related to the structure and function of silk fibroin in cartilage regeneration. The secondary outcomes we pursued included the potential for clinical translation and complications. Other variables we gathered include the author, title, year, publication year, journal, and country of publication.

#### 2.1.6. Data Synthesis and Statistical Analysis

Because the included studies differed substantially in scaffold composition, fabrication methods, experimental models, mechanical testing protocols, and reported outcomes, quantitative pooling and meta-analysis were not appropriate. Findings were therefore synthesized descriptively and organized according to study characteristics, mechanical design characteristics, mechanical and biological outcomes, and reported complications and translational barriers.

#### 2.1.7. NIH Quality Assessment

Study quality and risk of bias were assessed using the National Institutes of Health (NIH) quality assessment tool. Two reviewers independently evaluated each study and classified overall quality as poor, fair, or good.

## 3. Results

### 3.1. Study Characteristics

#### 3.1.1. Study Selection

A total of 24 studies published between 2014 and 2025 were included in this review. The bibliographic characteristics, study environments, and specific silk fibroin-based material compositions for each included article are summarized in Table 1.

#### 3.1.2. Study Settings and Models

Studies were conducted in China (44%), India (24%), Austria (8%), the Republic of Korea (8%), the USA (8%), Switzerland (4%), and Thailand (4%). The majority were in vitro studies (44%); a minority were in vivo (16%). In total, 40% of studies were combined in vivo/in vitro. One study was computational in nature. Rabbit models were the most prevalent (40%); mouse and human models were equal at 9% each. One study used a pig ear model (4%). Of note, one study combined human, rabbit, and mouse models (4%) and another study combined rabbit and mouse models (4%).

### 3.2. Mechanical Design Characteristics

#### 3.2.1. Scaffold Composition and Structural Reinforcement

All included scaffolds and hydrogels were composed of silk fibroin (SF) or an SF derivative. Early bioinks used unmodified or minimally processed SF, commonly blended with gelatin to obtain the viscosity required for extrusion-based printing [33]. Crosslinker-free silk–gelatin formulations used physical entanglement and beta-sheet formation for gelation, with gelatin serving as a bulking agent [24]. These formulations nevertheless exhibited low viscosity, slow gelation, and nozzle clogging [34]. Enzymatic crosslinking subsequently provided a biocompatible means of stabilizing SF–gelatin and SF–decellularized extracellular matrix (dECM) constructs, although control over gelation kinetics and mechanical properties remained limited [20,30,35].

Natural and synthetic components were incorporated to modify scaffold mechanics and biological function. Collagen was used in four studies [10,11,19,25], gelatin in nine [13,16,18,20,23,24,30,31,32], and both collagen and gelatin in two [9,12]. Hyaluronic acid was included in four studies to support hydration and elasticity [10,11,15,19]. Synthetic polymers were used in four studies, primarily polycaprolactone (PCL) and related polymeric reinforcements [14,21,23,26]. Two studies combined SF-based hydrogels with PCL to address osteochondral defects [21,26].

Chemical modification, particularly methacrylation, expanded control over gelation and construct geometry. Glycidyl methacrylate-modified SF enabled rapid photopolymerization [22], and methacrylated SF formulations supported digital light processing (DLP) of complex structures, including tracheal and ligament constructs [10,14,22]. SF methacrylate-polyethylene glycol diacrylate bioinks supported chondrocyte viability and cartilage-like matrix formation, including sulfated glycosaminoglycan and collagen type II deposition [23]. Double-network scaffolds combining SF with methacrylated hydroxypropyl methylcellulose were also used to increase elasticity and fracture resistance [27].

#### 3.2.2. Cellular and Bioactive Modifications

The scaffolds were enriched with mesenchymal stem or stromal cells [9,13,15,16,18,20,21,24,25,27,32,36,37] and chondrocytes [14,17,29,30,32,37]. Bioactive factors and small-molecule interventions were incorporated to influence chondrogenesis, inflammation, cell recruitment, and phenotypic stability. Covalent conjugation of small-molecule inhibitors and growth factors to SF–gelatin bioinks provided sustained delivery and supported phenotypic stability [18,36], while growth factor-rich plasma served as a source of transforming growth factor beta (TGF-beta) and connective tissue growth factor (CTGF) [16]. Functionalized scaffolds containing mechano growth factor and TGF-beta3 promoted endogenous stem cell recruitment and hyaline cartilage regeneration in rabbit osteochondral defect models [13,37].

#### 3.2.3. Fabrication Approaches

Extrusion-based bioprinting was the most common fabrication method. DLP was used in three studies, and one study reported 4D bioprinting [13]. Freeze-drying, casting, and finite element analysis modeling were also employed [9,11,12,15,26,31]. Compared with extrusion-based approaches, DLP of methacrylated SF enabled faster printing and higher-resolution structures while maintaining cell viability [38]. Hybrid fabrication combined fused deposition modeling with DLP to create mechanically stable tracheal constructs [10,14]. A 4D approach incorporated magnetic nanoparticles into SF–gelatin bioink to produce shape-morphing constructs responsive to external magnetic fields [13].

### 3.3. Mechanical and Biological Outcomes

#### 3.3.1. Mechanical Performance

Mechanical performance varied with scaffold composition, crosslinking, and processing. Early SF bioinks did not match the mechanical benchmarks of native articular cartilage [15]. A printed collagen type II/SF/hyaluronic acid scaffold combined high porosity with an increased compressive elastic modulus, although its mechanical performance remained below that of native tissue [11]. Across studies, beta-sheet content was a principal determinant of stiffness and stability [39], and double-network strategies improved elasticity and resistance to fracture [27]. Composite formulations containing hyaluronic acid increased hydration and elasticity, whereas PCL and polyethylene glycol derivatives provided structural reinforcement [10,11,21]. Mechanical properties were therefore tunable through composition, crosslink density, and beta-sheet crystallinity [23,25].

Direct quantitative comparison across studies was limited by heterogeneity in testing methods, including differences in hydration conditions and compression rates. The reported findings therefore support a descriptive synthesis of mechanical trends rather than a pooled comparison of compressive modulus, tensile properties, or viscoelastic behavior.

#### 3.3.2. Biological and Regenerative Performance

SF-based constructs supported chondrocyte viability, chondrogenic differentiation, and cartilage-like extracellular matrix formation across multiple formulations. Methacrylated SF–polyethylene glycol diacrylate constructs increased sulfated glycosaminoglycan and collagen type II deposition [23], while functionalized articular cartilage scaffolds promoted endogenous cell recruitment, host–tissue integration, and collagen organization resembling native cartilage in rabbit models [13,37]. Cell delivery also influenced performance: aggregate seeding on SF scaffolds more closely reproduced cell condensation, increased cell–cell interaction and differentiation, and reduced hypertrophy compared with dispersed-cell seeding [24].

Regenerative applications included articular cartilage, osteochondral repair, meniscus engineering, and tracheal reconstruction. PCL/SF implants designed for hip dysplasia supported both osteogenesis and chondrogenesis, with a reinforced layer providing mechanical support and an SF-based region supporting cartilage formation [21]. SF hydrogels containing dECM and gelatin reproduced aspects of meniscal architecture and supported differentiation of infrapatellar fat pad-derived mesenchymal stem cells [9,12]. Growth factor-rich plasma in SF bioinks provided sustained delivery of TGF-beta and CTGF and promoted meniscal matrix formation without chemical crosslinkers [16]. Hybrid SF constructs used for tracheal reconstruction maintained structural stability while supporting tissue formation [10,14].

### 3.4. Complications and Translational Barriers

Several studies reported biological, mechanical, and fabrication-related complications. Bioprinting was associated with early cell loss [14,20,23,28,32], including mortality of up to 46% within 24 h in one study [27]. Shear stress during extrusion and ultraviolet exposure during photocrosslinking were identified as potential contributors. Reported biological limitations also included inflammatory responses, tissue overgrowth, lumen stenosis, and the formation of fibrous rather than functional cartilage tissue in selected models [10,14,30].

Mechanical and manufacturing limitations included material fragility [40], construct instability beyond two weeks, stress concentrations in hybrid scaffolds [12], and shrinkage or distortion during culture or post-processing [9,12,20,25]. The low viscosity of SF complicated print fidelity and contributed to nozzle clogging or loss of shape, making gelatin blending or chemical modification necessary in many formulations. Hydrogel constructs also remained mechanically mismatched with native articular cartilage, with reported values often in the kilopascal range rather than the megapascal range of native tissue.

Degradation and material stability were not consistently synchronized with tissue formation. Gelatin and ECM components could degrade rapidly and permit early construct collapse, whereas highly crystalline or chemically crosslinked SF could persist and impede tissue integration. Swelling in physiological fluid could further reduce mechanical performance and alter construct shape. In vivo findings were also constrained by species-specific biomechanics and, in some models, unrestricted postoperative movement [21]. These limitations reduced the direct translational relevance of short-term preclinical outcomes.

## 4. Discussion

### 4.1. Mechanical Design Principles

The included studies indicate that the mechanical behavior of SF constructs is governed by the interplay among molecular structure, crosslinking strategy, composition, and scaffold architecture. Unmodified or lightly crosslinked SF hydrogels provide a compliant environment suitable for cell encapsulation but may lack sufficient mechanical support under load [9,11]. Increasing beta-sheet content through physical, enzymatic, or photo-crosslinking increases stiffness and structural stability [9,25,27,39], while crosslink density and crystallinity also affect degradation and swelling [23,25]. This relationship creates a central design tradeoff: stabilization can improve initial mechanical performance, but excessive crystallinity or crosslinking may slow degradation and interfere with tissue integration.

The mechanistic basis for this behavior lies in the molecular architecture of silk fibroin. The heavy chain contains repetitive hydrophobic domains rich in glycine–alanine repeats that serve as nucleation sites for β-sheet crystallization. These crystalline domains function as physical crosslinks, anchoring amorphous chain segments and resisting deformation under load [39]. Importantly, the relationship between crosslinking density and β-sheet content is nonmonotonic: Mao and colleagues showed that increasing chemical crosslinker concentration initially promoted β-sheet formation but suppressed it at higher concentrations, while the tensile modulus increased linearly and elasticity decreased. Excessive crosslinking also impaired degradation and provoked foreign body reactions in vivo [39]. Double network strategies further illustrate this complexity. Ni and colleagues created interpenetrating networks where covalently crosslinked HPMC-MA provided elasticity while physically crosslinked silk fibroin contributed stiffness through β-sheet formation [27], and Zhang and colleagues demonstrated that progressive β-sheet crystallization alone could achieve structural stability without any chemical crosslinker [25]. Together, these findings indicate that the structure–property relationships of SF scaffolds are determined by the combined effects of crosslinking type, crosslink density, network architecture, and polymer concentration rather than by any single parameter.

Composite and double-network designs further expand the range of achievable mechanical and biological properties. Gelatin increases viscosity and printability, hyaluronic acid supports hydration and elasticity, and collagen or dECM provides a more tissue-like biological environment. In contrast, PCL, polyethylene glycol derivatives, and methacrylated polymers provide reinforcement, rapid photopolymerization, or resistance to fracture [9,10,11,12,21,23,26,27]. Multilayer constructs extend this principle by assigning different mechanical and biological functions to discrete regions, including reinforced bone-facing layers and bioactive cartilage-facing layers [12,21,26].

Scaffold architecture also affects performance. Porosity facilitates cell distribution and transport but can reduce load-bearing capacity, whereas reinforced or multilayer geometries can distribute stress more effectively. The available mechanical data cannot be directly pooled because the hydration state, loading protocol, strain rate, construct geometry, and reported outcome measures differed among studies. Consequently, the evidence supports consistent structure–property trends but not a definitive ranking of formulations.

### 4.2. Engineering Strategies for Functional Cartilage Regeneration

Mechanical optimization alone did not determine regenerative performance. Most studies combined structural, cellular, biochemical, and fabrication strategies to compensate for the limitations of any single component. The progression from simple SF–gelatin blends to multicomponent and functionalized constructs therefore reflects an effort to balance printability, immediate stability, cell survival, tissue formation, and degradation.

#### 4.2.1. Structural and Composite Strategies

SF provided the structural foundation across all included studies. Collagen and dECM were used to create a more biologically representative extracellular environment, gelatin increased bioink volume and viscosity, and hyaluronic acid supported hydration and elasticity. Synthetic components served more explicitly mechanical functions: PCL and related polymers reinforced load-bearing constructs, while polyethylene glycol derivatives and methacrylation enabled tunable and rapid photocrosslinking [9,10,11,12,14,21,22,23,25,26,27]. In osteochondral constructs, combining an SF-based cartilage layer with a reinforced polymeric layer allowed the design to address cartilage and subchondral bone as mechanically and biologically distinct regions [21,26].

#### 4.2.2. Cell-Based Strategies

Chondrocytes and mesenchymal stem or stromal cells were used to populate the constructs and produce new extracellular matrix [9,13,14,15,16,17,18,20,21,24,25,27,29,30,32,37]. The method of cell delivery was also relevant: aggregate seeding reproduced aspects of developmental cell condensation and improved cell–cell interaction, differentiation, and hypertrophy control compared with dispersed-cell seeding [24]. These benefits must be balanced against early cell loss caused by extrusion shear stress or photocrosslinking exposure [14,20,23,27,28,32].

#### 4.2.3. Growth Factor Delivery and Chemical Functionalization

Growth factors and small molecules were incorporated to direct cell recruitment, chondrogenesis, inflammation, and phenotypic stability. Platelet-rich or growth factor-rich plasma acted as a reservoir for TGF-beta and CTGF [16], and functionalized constructs containing mechano growth factor and TGF-beta3 promoted endogenous cell recruitment and cartilage repair in rabbit osteochondral defects [13,37]. Because physical encapsulation may permit early burst release, covalent conjugation to the SF backbone was used to prolong localized delivery [18]. Affinity peptides, pathway inhibitors, anti-inflammatory molecules, and antioxidant additives were likewise used to recruit stem cells or suppress hypertrophic, inflammatory, and oxidative responses.

#### 4.2.4. Advanced Bioprinting and Modeling

Extrusion-based bioprinting remained the most common approach because it accommodates viscous, cell-laden materials, but its shear forces and limited shape fidelity contributed to cell loss and construct distortion. DLP offered faster printing and higher resolution for photo-crosslinkable SF derivatives [38], while hybrid fabrication paired polymer reinforcement with photocrosslinked biological regions [10,14]. Organ-on-chip systems enabled controlled evaluation of cellular responses to gradients and mechanical stress, and magnetically responsive 4D constructs introduced externally applied mechanical stimulation [13]. These methods expand design control but also increase fabrication complexity and the number of variables requiring validation.

Together, these approaches show that functional cartilage regeneration depends on coordinating scaffold mechanics with cell delivery, bioactive signaling, and fabrication. The same added complexity that improves biological performance, however, introduces additional barriers to reproducibility, long-term stability, and clinical translation.

### 4.3. Translational Challenges

#### 4.3.1. Biological Response and Phenotypic Stability

SF is valued for cytocompatibility and low immunogenicity, but SF alone does not reproduce the full biological signaling environment of native cartilage. Without dECM, peptides, growth factors, or optimized seeding, stem or stromal cells may produce fibrocartilage rather than stable hyaline cartilage. Printing-associated shear stress, ultraviolet exposure, and other crosslinking conditions can further reduce cell viability and retention [14,20,23,27,28,32]. These effects increase the number of cells required at implantation and may limit the efficiency and reproducibility of patient-derived cell strategies.

#### 4.3.2. Mechanical Mismatch and Fabrication Fidelity

Many hydrogel constructs remained substantially less stiff than native articular cartilage, with reported mechanical properties often in the kilopascal range rather than the megapascal range. Reinforcement and additional crosslinking can narrow this gap but may reduce biological permissiveness or alter degradation. Low SF viscosity also impairs extrusion fidelity and can cause nozzle clogging or loss of shape. Gelatin blending and methacrylation improve printability, yet shrinkage, swelling, dehydration, and post-crosslinking distortion can still produce a mismatch between the intended and final construct [9,12,20,25].

Furthermore, direct quantitative comparison of mechanical outcomes across studies was not feasible due to substantial heterogeneity in testing protocols, including differences in compression rates, hydration conditions, sample dimensions, and reporting units. This variability underscores the need for standardized biomechanical testing frameworks in future silk fibroin scaffold research to enable meaningful cross-study comparisons.

#### 4.3.3. Degradation and Long-Term Stability

An effective scaffold must retain sufficient mechanical function until newly formed tissue can bear load, and then degrade without obstructing integration. The reviewed formulations did not consistently achieve this balance. Rapid degradation of gelatin or ECM components could destabilize a construct, whereas highly crystalline or densely crosslinked SF could persist too long. Physiological swelling further altered geometry and mechanical properties. Most studies evaluated short-term outcomes, leaving limited evidence regarding fatigue resistance, viscoelastic performance, and shape retention over months of repeated loading.

#### 4.3.4. Preclinical Model Limitations

The evidence base was dominated by in vitro studies and small-animal models, particularly rabbits. Species-specific biomechanics and load-bearing conditions limit direct extrapolation to human cartilage, and unrestricted postoperative movement introduced an additional source of variability in some in vivo experiments [21]. The distinct mechanical requirements of articular cartilage, meniscus, trachea, and ligament-related constructs also prevent findings from being treated as interchangeable. Evidence from one application is therefore most informative when interpreted against the loading environment and function of that target tissue.

### 4.4. Clinical Implications

The findings support SF as a versatile platform rather than a single, universally applicable scaffold. For focal articular cartilage repair, hydrated and bioactive SF-based hydrogels may support chondrogenesis and host integration, but they require sufficient reinforcement for joint loading [11,13,15,37]. Osteochondral repair is better aligned with multilayer or hybrid constructs that separate the mechanical requirements of subchondral bone from those of the cartilage surface [21,26]. Meniscal applications require constructs that reproduce zonal architecture and resist complex tensile and compressive loading; SF hydrogels containing dECM, gelatin, or growth factor-rich plasma address the biological component of this problem but still require long-term mechanical validation [9,12,16].

Outside articular cartilage, hybrid SF constructs have been used for tracheal reconstruction, where maintenance of lumen geometry and resistance to stenosis are clinically important [10,14]. These studies demonstrate the adaptability of SF processing, but their outcomes should not be generalized directly to load-bearing joint cartilage. Similarly, mechanical reinforcement that is appropriate for ligament, tendon, or bone-facing regions may not reproduce the compliant, viscoelastic behavior required at a cartilage surface.

Clinical translation will therefore require indication-specific design. The most promising constructs are those in which material composition, architecture, degradation, cell source, bioactive delivery, and manufacturing method are selected for the mechanical and biological requirements of the target tissue rather than optimized in isolation.

### 4.5. Future Directions

Future studies should use standardized and transparently reported biomechanical testing to permit comparison across formulations. At minimum, testing conditions should specify the hydration state, construct geometry, loading direction, strain rate, and relevant compressive, tensile, or viscoelastic outcomes. Direct comparison with native cartilage and existing clinical standards would clarify the magnitude and clinical significance of mechanical mismatch.

Long-term studies are needed to determine whether scaffold degradation, extracellular matrix deposition, and restoration of mechanical function proceed at compatible rates. These studies should assess fatigue resistance, swelling, viscoelastic behavior, construct geometry, inflammatory response, and stability under repeated physiological loading rather than focusing only on early tissue formation [27].

A critical limitation of the current evidence base is the predominant reliance on rabbit and murine models. Rabbit cartilage is substantially thinner than human cartilage, and small animal models exhibit differences in load distribution related to quadrupedal gait and a greater capacity for spontaneous cartilage healing that cannot be extrapolated to human repair. Gao and colleagues acknowledged that unrestricted postoperative movement and species-specific biomechanics limited the translational relevance of their rabbit hip dysplasia model [21]. Future studies should prioritize validation in large animal models with cartilage thickness and joint loading conditions more closely approximating the human knee [37].

Translation also requires validation in models that more closely reproduce human joint size and loading. Large-animal studies, patient-specific construct designs, and explicit comparison of animal and human biomechanics would strengthen the interpretation of preclinical findings [37]. Because the reviewed applications have different mechanical requirements, future investigations should define the intended clinical indication and select outcome measures accordingly.

Finally, multicomponent bioinks should be evaluated as integrated systems. Combining SF with dECM, synthetic reinforcement, cells, and covalently bound bioactive factors may address multiple design requirements [18], but each additional component affects fabrication, mechanics, and degradation. Coordinating these variables through clinically driven design and reproducible testing will be necessary to advance SF-based constructs toward durable, functional cartilage repair.

## 5. Conclusions

This systematic review highlights silk fibroin as an adaptable biomaterial platform for cartilage regeneration due to its biocompatibility, tunable mechanics, and adaptability across advanced fabrication platforms. Across 24 studies, silk fibroin-based scaffolds demonstrated the capacity to support chondrogenesis when combined with appropriate structural reinforcement, bioactive cues, and cell-based strategies. However, persistent challenges, such as mechanical mismatch with native cartilage, fabrication-induced cell loss, phenotypic instability, and poorly synchronized degradation, limit current clinical translation. Addressing these gaps through standardized characterization, long-term in vivo validation, and clinically driven design will be essential for advancing silk fibroin-based constructs toward durable, functional cartilage repair.

## Figures and Tables

**Table 1:** Summary of included studies, authors, publication years, and scaffold composition.

Article Title	Authors	Publication Date	Country of Origin	Study Model	Scaffold Composition	NIH Quality Assessment
*Silk Fibroin-Based Hydrogels Supplemented with Decellularized Extracellular Matrix and Gelatin Facilitate 3D Bioprinting for Meniscus Tissue Engineering* [9]	Fritz et al.	June 2025	Austria	Rabbits	SF + tyrosine cross linking + intra-patellar fat pad mesenchymal stem cells (IFP-MSCs) + gelatin + DCM	Fair
*Construction of multifunctional tracheal substitute based on silk fibroin methacryloyl and hyaluronic acid methacryloyl with decellularized cartilaginous matrix for tracheal defect repair* [10]	Shan et al.	May 2025	China	Rabbits	SF + methacryloyl (SilMA) + hyaluronic acid methacryloyl (HAMA) + decellularized matrix (DCM)	Fair
*Mechanical properties and biocompatibility characterization of 3D printed collagen type II/silk fibroin/hyaluronic acid scaffold* [11]	Gao et al.	April 2025	China	Rabbits	Silk fibroin + collagen type II + hyaluronic acid (HA)	Good
*Biomechanical testing of virtual meniscus implants made from a bi-phasic silk fibroin-based hydrogel and polyurethane via finite element analysis* [12]	Moser et al.	February 2025	Austria	Virtual/ computational	Hydrogel—SF, gelatin (G), MD-dECM hybrid: Hydrogel Core/PU 54 or PU 205	Fair
*Protocol for developing shape-morphing 4D bioprinted magnetic constructs to promote articular cartilage regeneration using silk fibroin-gelatin bioink* [13]	Chakraborty et al.	December 2024	India	Rabbits	SF + gelatin + Fe3O4 magnetic nanoparticles	Good
*Hybrid 3D bioprinting for advanced tissue-engineered trachea: merging fused deposition* *modeling (FDM) and top–down digital light* *processing (DLP)* [14]	Lee et al.	November 2024	Republic of Korea	Rabbits	SF + SilMA + chondrocytes	Good
*Three-Dimensional Printed Silk Fibroin/Hyaluronic Acid Scaffold with Functionalized Modification Results in Excellent Mechanical Strength and Efficient Endogenous Cell Recruitment for Articular Cartilage Regeneration* [15]	Shi et al.	September 2024	China	Rabbits	Silk fibroin (SF) + methacrylated hyaluronic acid (MAHA) + E7 affinity peptide	Good
*Photo-Polymerizable Autologous Growth-Factor Loaded Silk-Based Biomaterial-Inks toward 3D Printing-Based Regeneration of Meniscus Tears* [16]	Bandyopadhyay et al.	May 2024	India	Rabbits	Silk fibroin + gelatin + autologous growth-factor rich plasma (GFRP)	Good
*An Anti-Oxidative Bioink for Cartilage Tissue Engineering Applications* [17]	Chen et al.	February 2024	China	Mice	Silk fibroin + methacrylate-modified rutin (RTMA)	Good
*Covalent Conjugation of Small Molecule Inhibitors and Growth Factors to a Silk Fibroin-Derived Bioink to Develop Phenotypically Stable 3D Bioprinted Cartilage* [18]	Majumder et al.	February 2024	USA	Humans	SF + gelatin + mushroom tyrosinase crosslinking	Fair
*Embedded Bioprinting of Breast Tumor Cells and Organoids Using Low-Concentration Collagen-Based Bioinks* [19]	Shi et al.	October 2023	USA	Mice	SF + low-concentration collagen	Good
*Development of a biomimetic arch-like 3D bioprinted construct for cartilage regeneration using gelatin methacryloyl and silk fibroin-gelatin bioinks* [20]	Chakraborty et al.	April 2023	India	Humans	SF + gelatin methacryloyl	Good
*3D-printed regenerative polycaprolactone/silk fibroin osteogenic and chondrogenic implant for treatment of hip dysplasia* [21]	Gao et al.	December 2022	China	Rabbits	PCL + SF	Good
*Tissue engineering approaches for the repair and regeneration of the anterior cruciate ligament: towards 3D bioprinted ACL-on-chip* [22]	Bakirci et al.	August 2022	Switzerland	Rabbits, mice	SF + Hyaluronic acid + alginate + collagen + GeIMA + polyethylene glycol	Fair
*Advanced 3D-Printing Bioinks for Articular Cartilage Repair* [7]	Liang et al.	April 2022	China	Humans, rabbits, mice	Silk fibroin	Fair
*3D bioprinting of photo-crosslinkable silk methacrylate (SilMA)-polyethylene glycol diacrylate (PEGDA) bioink for cartilage tissue engineering* [23]	Bandyopadhyay et al.	April 2022	India	Pig ear cartilage	SF + PEGDA + gelatin + LAP + protease and collagenase + phosphate-buffered saline + chondrocytes	Fair
*3D printed silk-gelatin hydrogel scaffold with different porous structure and cell seeding strategy for cartilage regeneration* [24]	Li et al.	October 2021	China	Rabbits	SF + gelatin-tyramine	Fair
*Crosslinker-free silk/decellularized extracellular matrix porous bioink for 3D bioprinting-based cartilage tissue engineering* [25]	Zhang et al.	January 2021	China	Mice	SF-dECM + bone marrow mesenchymal stem cells (BMSCs)	Fair
*The 3D-Printed Bilayer’s Bioactive-Biomaterials Scaffold for Full-Thickness Articular Cartilage Defects Treatment* [26]	Thunsiri et al.	August 2020	Thailand	In vitro	SF + PLA (polylactic acid), PCL (polycaprolactone), CS (chitosan), and HA (hydroxyapatite)	Fair
*3D Bioprinting of Bone Marrow Mesenchymal Stem Cell-Laden Silk Fibroin Double Network Scaffolds for Cartilage Tissue Repair* [27]	Ni et al.	August 2020	China	In vitro	SF + hydroxypropyl methyl cellulose of methacrylation (HPMC-MA)	Fair
*Addition of Platelet-Rich Plasma to Silk Fibroin Hydrogel Bioprinting for Cartilage Regeneration* [28]	Li et al.	August 2020	China	In vitro	SF/PEG and SF-PRP/PEG	Fair
*Digital light processing 3D printed silk fibroin hydrogel for cartilage tissue engineering* [29]	Hong et al.	February 2020	Republic of Korea	Rabbits	SF + methacrylate (silk-GMA) crosslinked with DLP	Good
*3D Bioprinting Using Cross-Linker-Free Silk-Gelatin Bioink for Cartilage Tissue Engineering* [30]	Singh et al.	September 2019	India	Mice	SF+ gelatin	Fair
*Structurally and Functionally Optimized Silk-Fibroin-Gelatin Scaffold Using 3D Printing to Repair Cartilage Injury* In Vitro *and* In Vivo [31]	Shi et al.	August 2017	China	Rabbits	SF + gelatin + conjugated E7 peptide	Good
*Regulation of Chondrogenesis and Hypertrophy in Silk Fibroin-Gelatin-Based 3D Bioprinted Constructs* [32]	Chameettachal et al.	September 2016	India	Humans	SF + gelatin + tyrosinase cross-linking	Good

*SF: Silk fibroin. DCM: Decellularized matrix. silk-GMA: Silk fibroin-glycidyl methacrylate. PCL: Polycaprolactone. GelMA: Gelatin methacrylate. PEGDA: Polyethylene glycol diacrylate. PEG: Polyethylene glycol. PRP: Platelet-rich plasma. DLP: Digital light processing*.

## Data Availability

No new data were created or analyzed in this study. Data sharing is not applicable to this article.

## Supplementary Materials

The following are available online at https://www.mdpi.com/article/10.3390/biomimetics11080575/s1, Figure S1: PRISMA flow diagram illustrating the identification, screening, eligibility assessment, and inclusion of studies in this systematic review.
