# Supplementary material for: Biomechanical Properties of Silk-Derived 3D Bioprinted Scaffolds for Cartilage Regeneration: A Comprehensive Review
Source: Biomimetics (Basel). 2026 Aug 12;11(8):575. doi: 10.3390/biomimetics11080575 (PMC13511671; doi:10.3390/biomimetics11080575)
Supplement: Supplementary file 1 [file biomimetics-11-00575-s001.zip › biomimetics-4383228-supplementary.pdf]

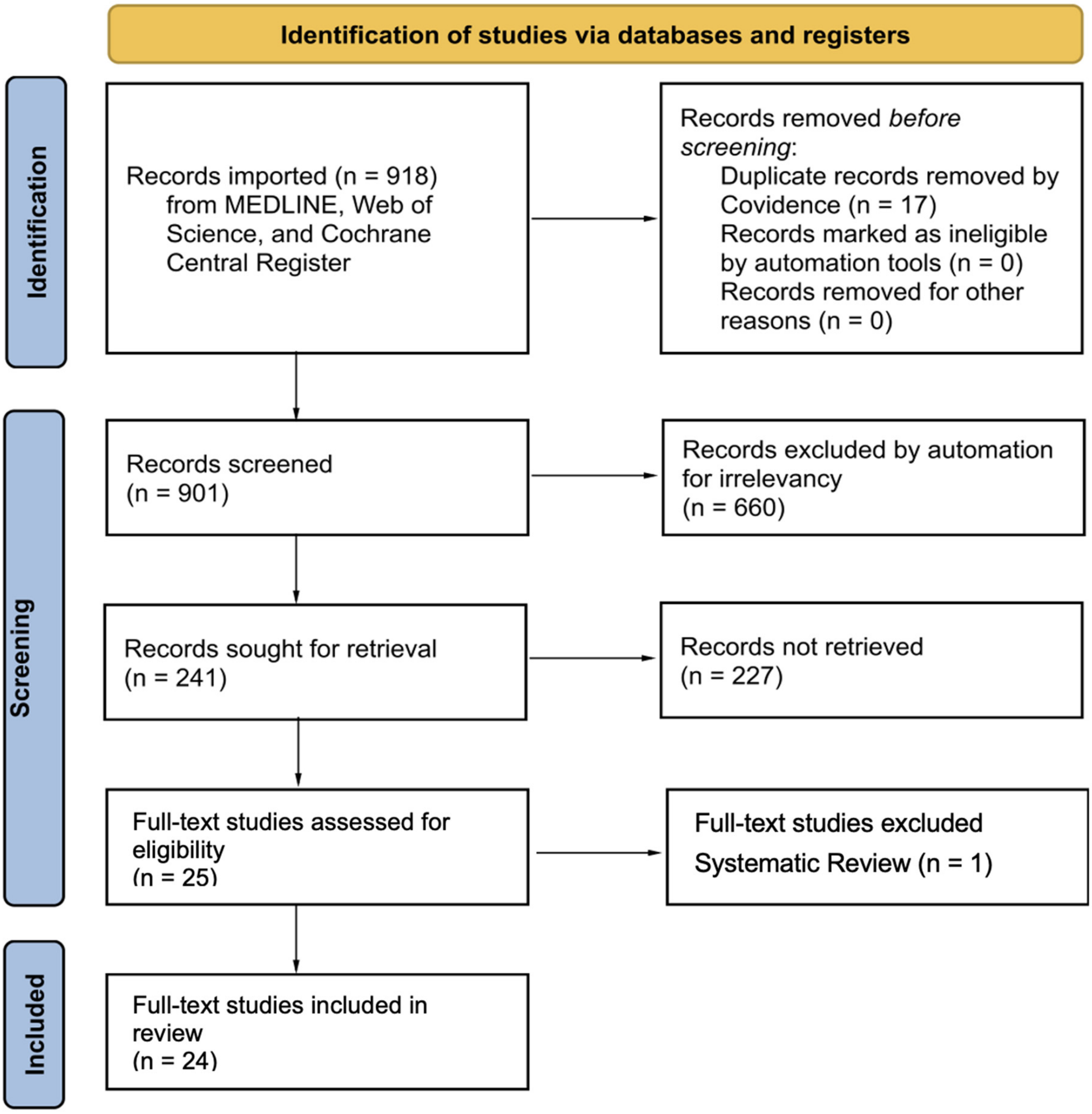

Figure S1. PRISMA flow diagram illustrating the identification, screening, eligibility assessment, and inclusion of studies in this systematic review.
